# Immersive psychiatry simulation: a novel course for medical student training

**DOI:** 10.1192/bjo.2021.430

**Published:** 2021-06-18

**Authors:** Kenneth Ruddock, Kim Herbert, Catriona Neil, Neera Gajree, Karli Dempsey

**Affiliations:** NHS Lanarkshire

## Abstract

**Aims:**

Over the last decade, there have been significant developments in the use of simulation for undergraduate medical education. Despite simulation's diverse applications across the medical school curriculum, it has thus far been underutilised within psychiatry teaching. Psychiatric simulation can support students to develop strategies to elicit psychopathology, de-escalate an aggressive patient or perform a risk assessment. Such experiences can be difficult to encounter during clinical placements and may expose a student or patient to an unacceptable level of risk. We have therefore developed an immersive simulation course that aims to enhance undergraduate psychiatry training.

**Method:**

Our course was developed by medical education faculty and psychiatry staff. The course handbook includes storyboards, patient scripts and debrief guidelines. Clinical scenarios are mapped to university intended learning outcomes and include; conducting a risk assessment for a patient with emotionally unstable personality disorder and comorbid depression, managing a manic patient in the Emergency Department and assessing a patient with obsessive-compulsive disorder who has developed skin damage due to hand washing.

The one-day course is delivered to groups of 4-8 students from the Universities of Glasgow and Edinburgh during their placements in NHS Lanarkshire. The course takes place in a simulation suite and is facilitated by psychiatrists and medical education faculty. Students each take the lead role during a clinical scenario in which they will encounter a simulated patient. Live video from the simulation is broadcast to other candidates. Scenarios last 10-15 minutes with a 20-30 minute group debrief immediately afterwards. The debrief utilises the PEARLS framework (Promoting Excellence and Reflective Learning in Simulation) and provides the opportunity for peer and facilitator feedback, as well as discussions regarding mental state examination, diagnosis and management.

**Result:**

Qualitative and quantitative feedback has been collected via an anonymous electronic post-course questionnaire. To date, the course has received universally positive feedback. 93% of candidates rated the overall quality of the course as a learning experience as ‘excellent’. Students reported that the course helped them develop communication skills which they could apply to future clinical situations. In addition, candidates felt participation had increased their confidence in taking a psychiatric history and performing a risk assessment.

**Conclusion:**

Immersive simulation is an underutilised tool in psychiatry education. Our course complements the existing educational programme of lectures and ward-based teaching and has been positively received. It provides the opportunity for students to develop interview techniques and communication skills in a safe, controlled environment.

